# The Accuracy of Imminent Death Diagnosis in a Palliative Care Setting

**DOI:** 10.7759/cureus.9503

**Published:** 2020-08-01

**Authors:** Abdullah I Alsuhail, Balaji Punalvasal Duraisamy, Abdullah Alkhudhair, Sami A Alshammary, Abdullah AlRehaili

**Affiliations:** 1 Palliative Care, King Fahad Medical City, Riyadh, SAU; 2 Radiation Oncology and Palliative Care, King Fahad Medical City, Riyadh, SAU; 3 Family Medicine, King Fahad Medical City, Riyadh, SAU

**Keywords:** prognostication, imminent death, end of life, palliative care

## Abstract

Background

Prognostication is important for patients and their family members as they need this information for the preparation and planning of their future. It is important for physicians as they desire to be accurate in their prognostication skills in order to plan and deliver better care to their patients; healthcare managers require it as they need this information for planning and distribution of hospital resources. We intended to study the accuracy of imminent death diagnosis (IDD) in a palliative care setting in all patients who died at the Palliative Care Unit (PCU) at King Fahad Medical City between December 2012 and December 2014.

Methods

We conducted a retrospective chart review of all consecutive patients who died in the PCU between 2012 and 2014. We studied the percentage of patients who were diagnosed with imminent death. We further looked at the accuracy of IDD by calculating the time between the diagnosis of imminent death and death. The primary outcomes were the percentage of patients who had an IDD and the proportion of those who died within 14 days of IDD. The secondary outcomes were the difference between patients who die after IDD and patients who die without imminent death diagnosis (NIDD) at the end of life interventions.

Results

During the period from December 2012 until December 2014, 48 patients died in the PCU. The majority of 28/48 (58%) died with IDD. However, 20/48 (42%) died NIDD. In the IDD group, 25/28 (89.3%) died within 14 days of diagnosis while 3/28 (10.3%) died after 14 days

Conclusions

IDD is a critical skill for palliative care physicians to make an advance care plan. Our study showed a high degree of accuracy of prediction of fourteen-day mortality in PCU patients. The median survival was two days. However, a large proportion of patients still died without a documented IDD. Multidisciplinary team input improves the accuracy of IDD. We recommend further studies be done to explore how IDD could improve care planning for dying patients and families.

## Introduction

Prognostication or estimation of time of death is significant [1-5] for patients and their family members alike. This information is vital for them to plan and prepare for their future. Physicians desire to be accurate in their prognostication skills in order to plan and deliver better care to their patients and the healthcare managers need this information for planning and distribution of hospital resources.

There are many prognostic tools used widely in palliative care by physicians for the estimation of prognosis [1]. Palliative Performance Scale (PPS), Palliative prognostic score (PaP), Palliative Prognostic Index are a few examples. However, the most commonly used method is by clinicians’ impression based on examination - clinical prediction of survival.

The European Association for Palliative Care (EAPC) suggests that clinical prediction of survival is a valid tool to obtain a general prognostication of patients’ survival, but it is subject to several factors like subjective variations and inter-observer variations, that limits its accuracy. The EAPC further recommends the use of clinical signs like performance status, anorexia-cachexia syndrome, dyspnoea, cognitive failure or delirium along with laboratory features like leucocytosis, lymphocytopenia, high C-reactive protein for prognostication at the end of life (with a word of caution about relying too much on lab investigations) [6-8]. The prognostic tools include all of the above clinical and laboratory features with few exceptions.

While it is usually recommended to use validated prognostic tools for prognostication of life expectancy, it is not widely practiced, and physicians tend to rely more on clinical predictions. It is worthwhile to note that there are no impact studies to prove that the routine use of prognostic tools in palliative care improves clinical, patient-related outcomes [6,8]. Further, in one study, multivariate regression analysis shows that model tools that included physicians' prognostic estimates were more accurate than models without physician input [9]. While it is true that statistical models can be more accurate than human intuition alone, it is also true that physicians provide critical information from clinical examination and experience that is seldom captured in statistical models alone.

It is known that health professionals tend to over-estimate or under-estimate prognosis. Nevertheless, it is important to note that the physician’s clinical estimate correlates with actual survival [8]. It is noteworthy that the more experienced doctors were more accurate in prognosticating survival [10]. It is also interesting to note that other studies have found the accuracy to be maximum in the last two weeks of life or close to death [8,11]. For example, in a prospective cohort study of 504 terminally ill patients and their 365 doctors found that only 20% of the doctors' predictions were accurate: 63% were optimistic (overestimated), and 17% pessimistic (underestimated) [11].

Further, prognostic accuracy improves with repeated re-assessments by the physician [12]. Because palliative care physicians spend more time with patients who are at the end of life and usually assess and reassess for signs of imminent death, it is possible that they are more accurate than what is stated in the literature [11]. In a study done in the UK to estimate the accuracy of prognostication by palliative care physicians, the accuracy of prediction of 14-day mortality was 70% [11]. We intended to explore this by conducting a study to evaluate the current level of accuracy of palliative care physicians practicing the imminent death protocol in our hospital.

The palliative care unit (PCU) at Cancer Center of King Fahd Medical City (KFMC) was established in December 2012. The imminent death protocol was developed for very sick patients who were expected to survive for less than two weeks.

We have a multidisciplinary team of physicians, nurses, physical and occupational therapists, dieticians, pharmacists, social workers, psychologists, and chaplains. We have a weekly multidisciplinary meeting as well as routine assessment by most of the team members. The imminent death diagnosis (IDD) was made by two physicians along with the multidisciplinary team.

The Oxford Textbook of Palliative Medicine defines the terminal phase as “The period of inexorable and irreversible decline in functional status before death.” The terminal phase or the phase of imminent death lasts from 24 hours to ≈14 days. It has three stages [13]:

Early

Bedbound

Loss of interest and/or ability to drink/eat

Cognitive changes: increasing time spend sleeping and/or delirium

Middle

Further decline in mental status to obtundation (slow to arouse with stimulation; only brief periods of wakefulness)

Late

Death rattle - pooled oral sections that are not cleared due to loss of swallowing reflex

Coma

Fever - usually from aspiration pneumonia

Altered respiratory pattern - periods of apnea, hyperpnea, or irregular breathing

Mottled extremities."

The diagnosis depends on the above physical signs such as reduced oral intake and decreased level of consciousness, death rattle, dropping nasolabial fold, cold extremities, mottled skin, and vital signs disturbance.

The imminent death protocol starts with documenting the diagnosis in the chart with date and time, then conducting a family meeting with the family. The physician has to fill up the checklist orders which contains several orders such as transferring patient to single bed, open visiting hours, allowing two watchers, referral to social, psychological and spiritual services, discontinuing unnecessary medications, adding and changing route/dose of the medications that are needed to control the most common symptoms at end of life like pain, dyspnea, death rattle and terminal agitation.

Objective

The study aims to evaluate the current situation regarding the accuracy of palliative care physicians’ clinical predictions of time of death from initiation of imminent death protocol. The research hypothesis suggests that patients will die within 14 days' post IDD.

Outcomes

The primary outcome is the percentage of IDD who died within 14 days divided by IDD who died after that and the median survival after IDD. The secondary outcome is the difference between patients who die after IDD and patients who die without imminent death diagnosis (NIDD) in many end of life interventions such as dying in a single bed, discontinuation of labs and vital signs, given artificial hydration and nutrition and given antibiotics, anticoagulation, and end of life medications.

## Materials and methods

Study population

All patients who died in the PCU between 2012 and 2014 at KFMC, Riyadh.

Study type

Retrospective cohort study by chart review. The study was approved by the Institutional Review Board.

Statistical analysis

All categorical variables like gender, age group, vitals, lab, etc. were presented as numbers and percentages. Continuous variables like age, IDD time to death, oxygen saturation, etc. were expressed as mean ± SD. Pearson's chi-square/Fisher’s exact test was used according to whether the cell expected frequency is smaller than 5, and it was used to determine the significant relationship between categorical variables. P - the value of less than 0.05 was considered as statistically significant. All data was entered and analyzed through Statistical Package for the Social Sciences (SPSS), version 22.0 (IBM Corp., Armonk, NY).

## Results

During the period from December 2012 until December 2014, 48 patients died in the PCU. The majority 28/48 (58.3%) died with IDD. However, 20/48 (41.7%) died NIDD. In the IDD group, 25/28 (89.3%) died within 14 days of diagnosis while 3/28 (10.7%) died after 14 days. The ratio of inaccurate diagnosis to accurate diagnosis is one to 8 (Table 1).

**Table 1:** Survival accuracy IDD: imminent death diagnosis; NIDD: no imminent death diagnosis

Patients death status	Patients died with diagnosis (IDD)	28 (58.3%)
	Patients died without diagnosis (NIDD)	20 (41.7%)
Estimated survival in days for IDD group	Time between IDD and death within 14 days (accurate diagnosis)	25 (89.3%)
	Time between IDD and death after 14 days (inaccurate diagnosis)	3 (10.7%)

The duration between imminent death protocol initiation and death was documented for all IDD group for any death within 14 days (336 hours) is considered as accurate death estimation. The average interval between IDD and death was 141 hours (confidence interval (CI) 31-251) which is equal to six days. However, because the survival data is not normally distributed, we rely more on the median. The median survival is 50 hours (CI 35-85) which is equal to two days. Nevertheless, one of the patients died after 1425 hours of initiation IDD. If we exclude this outlier, then the mean reduces to 93.5 hours (CI 40-146) and median 46 (CI 34-83). The mean of accurate survival group is 59 hours (CI 39-78), and median survival is 44 (CI 32-72) (Table 2).

**Table 2:** Time between imminent death diagnosis and death (hours)

	Number	Mean (CI*)	Median (CI)	Range
All IDD	28	141(31-251)	50 (35-85)	7-1425
Exclude outlier**	27	93.5 (41-146)	46 (34-83)	7-550
Only accurate***	25	58.7(39-78)	44 (32-72)	7-184

The total of 17 males and 31 females was included in the study with average age 59 years (SD 18). All the subjects are cancer patients with a variety of diagnosis (Table 3).

**Table 3:** Demographic data of the population: gender, age, and diagnosis IDD: imminent death diagnosis; NIDD: no imminent death diagnosis

Characteristics	IDD	NIDD	Total
Gender	Male 10(35.7%)	Male 7 (35%)	Male 17 (35.8%)
	Female 18 (64.3%)	Female 13 (65%)	Female 31 (64.6%)
Age group			
<45 year	5 (18%)	4 (20%)	9 (18.8%)
>45 year	23 (82%)	16(80%)	39 (81.3%)
Diagnosis			
Brain tumour	2	1	3
Other rare tumors	5	6	11
Head and neck	2	1	3
Lung cancer	0	1	1
Breast cancer	2	1	3
Gynaecological tumour	3	3	6
Genitourinary tumor (include Prostate cancer)	4	0	4
Haematological disease	0	2	2
Gastrointestinal tumours	10	5	15

**Figure 1:**
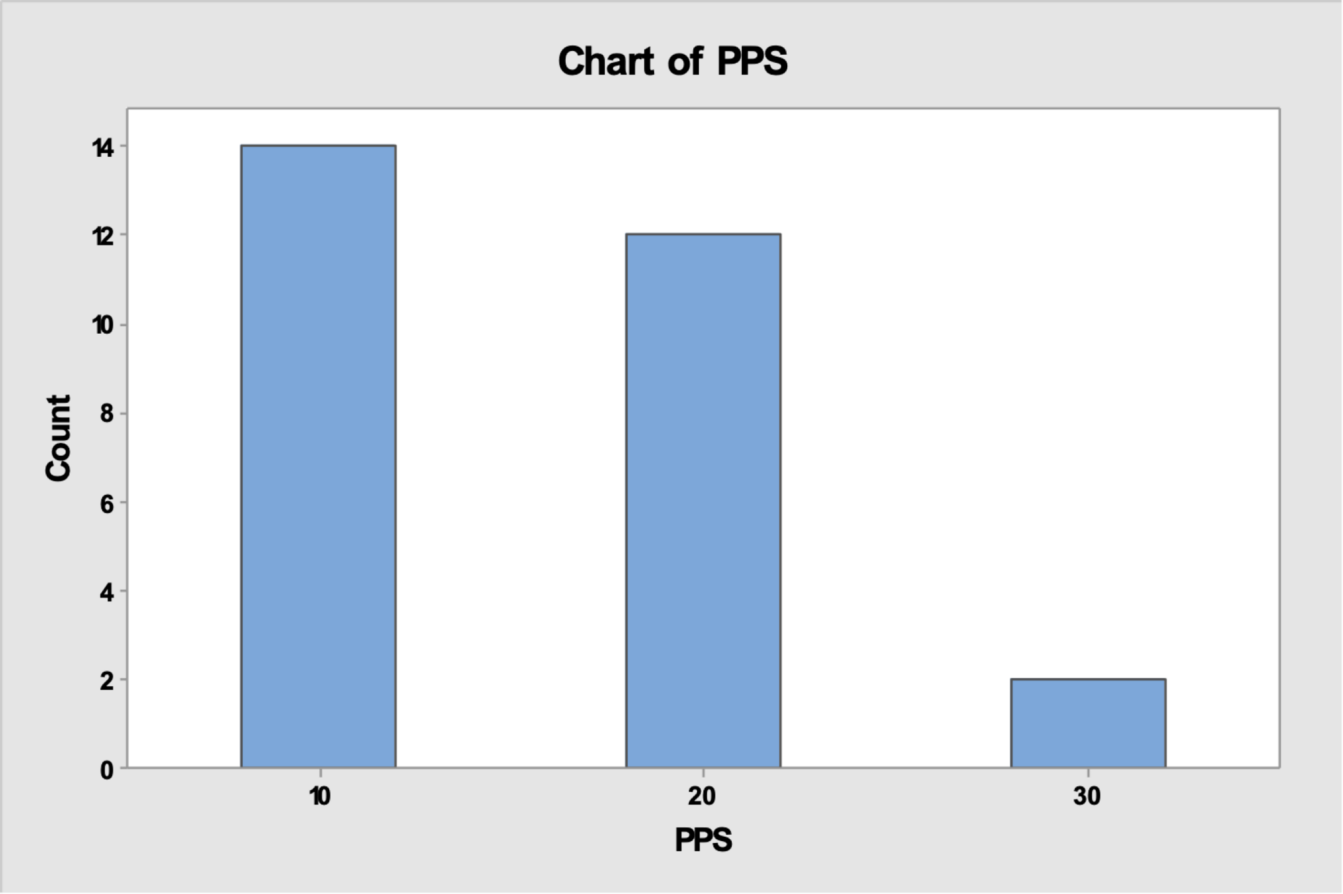
Palliative performance score (PPS) at time of imminent death diagnosis

The PPS at IDD was 10% in 14 patients, 20% in 12 patients and 30% for only two patients (Figure 1).

The prognosis depends on the functional status, impaired cognition, poor appetite, and labs. The main laboratory features of imminent death are leucocytosis, lymphocytopenia, and hypoalbuminemia. The data showed that there is no difference between IDD group and NIDD group in those five predictors (Table 4).

**Table 4:** Prognostic lab differences between IDD group and NIDD According to KFMC lab reference: *Albumin level 35-50, any level less than 35 is considered as hypoalbuminemia. **Neutrophils normally between 30%-70% of white blood count and normal absolute neutrophils count between 1.35-7.5*109/L ***Lymphocyte normally between 23%-60% of white blood count and the absolute lymphocyte count between 1.5-4.3 109/L IDD: imminent death diagnosis; NIDD: no imminent death diagnosis

	IDD	NIDD	P-value
Albumin* more than 35 mg/dl	0	3	0.079
less than 35 mg/dl	28	17	
Neutrophils** Less than 70%	6	7	0.765
More than 70%	22	13	
Absolute Neutrophils Less than 7.5*10^9^/L	12	10	0.624
More than 7.5*10^9^/L	16	10	
Lymphocyte*** Less than 23%	2	2	0.560
More than 23%	26	18	
Absolute lymphocyte Less than 1.5*10^9^/L	6	6	0.462
More than 1.5*10^9^/L	22	14	

Those patients who were diagnosed with IDD had more chance to discontinue unnecessary lab investigation, and vital signs compared to NIDD group. Also, patients with IDD were given artificial nutrition more than those in NIDD group. However, no difference in other intervention such as dying in a single bed (private room), artificial hydration, antibiotics or anticoagulation (Table 5). 

**Table 5:** The difference between IDD group and NIDD group in end of life care practice IDD: imminent death diagnosis; NIDD: no imminent death diagnosis

	IDD	NIDD	P-value
Died in single room Yes/No	17/11	9/11	0.284
Lab discontinue before death Yes/No	13/15	2/18	0.005*
Vital sign discontinues before death Yes/No	16/12	1/19	<0.001
Artificial hydration continues until death Yes/No	24/4	14/6	0.186
Artificial nutrition continues until death Yes/No	8/20	0/20	0.039*
Antibiotics given until death Yes/No	16/12	12/8	0.843
Anticoagulation continue until death Yes/No	13/15	7/13	0.428

No differences were observed in the opioid prescription between the IDD and NIDD group. Nevertheless, IDD group received more haloperidol and glycopyrrolate than the NIDD group (Table 6).

**Table 6:** The difference between IDD and NIDD in receiving the end of life medications IDD: imminent death diagnosis; NIDD: no imminent death diagnosis

	IDD	NIDD	P-value
Opioid Yes/ No	22/ 6	15/ 5	0.772
Haloperidol Yes / No	24/ 4	11/ 9	0.018*
Glycopyrrolate Yes / No	23/ 5	6/ 14	<0.001**

## Discussion

Patients with incurable, progressive, terminal illnesses deteriorate due to the progression of their disease and become imminently dying towards the last phase of life. The process of dying in the last phase is irreversible and is usually characterized by clinical signs that are important to make a diagnosis of imminent death. Once the dying process starts in a terminally ill patient, death is not preventable and usually occurs in a matter of days to weeks [14].

The diagnosis of imminent death is more important than being accurate about the time frame (similar to diagnosing labor) because the diagnosis is followed by irreversible physiological changes leading to death. The family and the patient have different needs and priorities during this period, and the palliative care team should make all efforts to support the patient and the family during this crucial phase of dying [15].

In our study, the majority of the patients died with a diagnosis of imminent death 58% (28). We used clinical examination, palliative performance status, and clinician prediction to arrive at the diagnosis of imminent death. As mentioned in the introduction and methods, we used a multidisciplinary approach to arrive at IDD. This approach probably resulted in a more accurate diagnosis of imminent death with a median survival of two days and a 90% accurate prediction of 14-day mortality. However, the multidisciplinary approach is also time consuming as we had to wait for all members to be involved in the decision making process (decision to initiate imminent death protocol). This could have been one of the reasons why 42% (20) of patients died NIDD.

In a different study published from our center, we found that the median interval between first palliative care consultation and death was 19 days [16]. Late referrals to palliative care lead to hesitations in making a diagnosis of imminent death. The patient is not well known to the members of the palliative care multidisciplinary team, who have difficulty with differentiating reversible causes of physiological decline like sepsis from imminent death. Further, the short time frame prevents the team from forming a trust-based relationship with the patient and family member. Such challenges also hinder effective communication between the patient, family, and palliative care team.

It is difficult to compare the survival of patients with IDD as opposed to those with NIDD due to the wide variation in the duration from the start of transfer to the PCU to death. The average mortality in the PCU (unpublished hospital data) is about 50%. This means that the remaining patients are discharged to home/hospice and so, diagnosing imminent death becomes all the more significant as it changes the advance care plan for these patients.

One of the important interventions upon diagnosing imminent death is shifting the patient to a single room. Table 5 shows that some of the patients (11) were not shifted to a single room due to non-availability. While 11 other patients who were not diagnosed to be imminently dying, died in single rooms. The single room gives much-needed privacy and facilitates us to open the visiting hours for the dying patients and their families.

The imminent death protocol and the end of life care policy were conceived during the inception of the PCU. Ideally, no patient under palliative care having a goal of comfort care till the end of life in the hospital should have had routine labs, insulin-glucose-check, and artificial feeding. Our study was more an audit of prevailing practice in 2012-2014. The results describe the available laboratory reports for the patients. No significant finding to assist in IDD could be found. This is in agreement with the international guidelines that IDD can be made effectively using clinical examination along with standardized prognostication tools, without any laboratory investigations.

We noted that patients with IDD had significantly more artificial feeding and hydration compared to those with NIDD. One of the early signs of imminent death is dysphagia. As already noted, because the end of life care policy was still being developed in 2012, the practice was to continue the artificial feeding, usually started by the oncologist before the transfer of care to palliative. The late referrals would make it more challenging to convince the family about withholding/withdrawing artificial nutrition.

However, those with IDD were significantly more likely to get the laboratory investigations and vital signs monitoring discontinued compared to those who were not diagnosed with imminent death.

There was no significant difference in the usage of opioids in both the groups. This is expected as every patient in palliative care would have received opioids for control of pain and dyspnea, irrespective of whether they were imminently dying or not. However, those with a diagnosis of imminent death were more likely to receive haloperidol for terminal agitation and glycopyrrolate for terminal secretions/death rattle.

A large number of patients (28) were given antibiotics till death, irrespective of whether or not imminent death was diagnosed. In an acute inpatient palliative care setting, with a challenge of late referrals, most patients have infections and sepsis, and as a policy, we treat infections in inpatients. Some of these patients had antibiotics started by the oncology team for neutropenia and sepsis and withdrawing those antibiotics is not agreeable to most patients and family members.

It is difficult to compare our study with other studies because we depended on clinical prediction and PPS to diagnose imminent death, instead of standardized prognostication tools. However, vast literature is available on the signs that occur during the last days of life [17]. Decreased level of consciousness, PPS ≤20%, and dysphagia of liquids appear > three days before death. Apnea periods, Cheyne-Stokes breathing, death rattle, peripheral cyanosis, pulselessness of radial artery, respiration with mandibular movement, and decreased urine output occurred mostly in the last three days of life. We relied on PPS and most of the above clinical signs to arrive at a diagnosis of imminent death.

The median survival for patients with PPS 30% is 7-37 days, PPS <20% is 1-9 days [18]. In our study, 90% of patients died within 14 days, and the median survival was about two days.

Strengths and limitations

Our study was a small retrospective study involving only cancer patients. We did not use standard prognostication tools. Continuing artificial nutrition and laboratory investigations for a patient under palliative care are not in line with international standards of care.

However, we were accurate in identifying and diagnosing imminent death in the majority of the patients. We had a standard multidisciplinary approach to arrive at this diagnosis. We could achieve this in spite of challenges like late referrals and during the early days of starting PCU in the center.

Based on the findings of this study, we had made recommendations to the administration to facilitate early referrals to palliative care and to convert all shared rooms in the inpatient PCU to private rooms. Both these were achieved in early 2017.

## Conclusions

Making an IDD is a unique and crucial skill that palliative care physicians have to develop for efficient advance care planning. Our study showed a high degree of accuracy of prediction of two-week mortality in cancer patients under palliative care. The median survival of the patients with an IDD was two days. However, an official IDD was not made in a large proportion of patients. We conclude that a multidisciplinary approach to IDD is more accurate in spite of being more time consuming. We recommend further studies to explore how IDD could improve care planning for dying patients and their families.
